# Are Microplastics Toxic? A Review from Eco-Toxicity to Effects on the Gut Microbiota

**DOI:** 10.3390/metabo13060739

**Published:** 2023-06-09

**Authors:** Huixia Niu, Shaojie Liu, Yujie Jiang, Yang Hu, Yahui Li, Luyang He, Mingluan Xing, Xueqing Li, Lizhi Wu, Zhijian Chen, Xiaofeng Wang, Xiaoming Lou

**Affiliations:** 1 Health Science Center, Ningbo University, Ningbo 315000, China; 2111101061@nbu.edu.cn (H.N.); 2111101059@nbu.edu.cn (Y.J.); 2011101047@nbu.edu.cn (Y.H.); 2 Department of Urology, Xijing Hospital, Air Force Medical University, Xi’an 710032, China; liusj1013@fmmu.edu.cn; 3 Zhejiang Provincial Center for Disease Control and Prevention, 3399 Binsheng Road, Hangzhou 310051, China; 881012022129@hmc.edu.cn (Y.L.); 202211125811023@zcmu.edu.cn (L.H.); mlxing@cdc.zj.cn (M.X.); xqli@cdc.zj.cn (X.L.); lzhwu@cdc.zj.cn (L.W.); zhjchen@cdc.zj.cn (Z.C.); xfwang@cdc.zj.cn (X.W.)

**Keywords:** micro- and nanoplastics, microplastics, polystyrene, toxicity, gut microbiota

## Abstract

Emerging studies have presented an initial picture of the toxic effects of exposure to environmental micro- and nanoplastics. They have indicated that micro- and nanoplastics may induce toxicity by leading to oxidative stress, energy metabolism disorders, gene damage, and so forth in environmental organisms, marine invertebrates and vertebrates, and laboratory mouse models. In recent years, micro- and nanoplastics have been discovered in human fecal samples, placentas, lung tissue, and even blood; thus, micro- and nanoplastics pose an alarming and ever-increasing threat to global public health. However, current research on the health effects of micro- and nanoplastics and the possible adverse outcomes in humans has only presented the tip of the iceberg. More robust clinical data and basic experiments are still warranted to elucidate the specific relationships and mechanisms. In this paper, we review studies on micro- and nanoplastic toxicity from the perspectives of eco-toxicity, the adverse effects on invertebrates and vertebrates, and the impact of micro- and nanoplastics on the gut microbiota and its metabolites. In addition, we evaluate the toxicological role of micro- and nanoplastic exposure and its potential implications in respect to human health. We also summarize studies regarding preventive strategies. Overall, this review provides insights on micro- and nanoplastic toxicity and its underlying mechanisms, opening up scientific avenues for future in-depth studies.

## 1. Background

Micro- and nanoplastics pollution has attracted considerable attention from the international scientific community, and its impact on human health and its associated mechanisms have emerged as leading research frontiers. In 2016, the United Nations Environment Assembly identified marine plastic debris and microplastics as significant global environmental issues. Coinciding with the widespread production and utilization of plastic over the past century, the global annual production of plastic in 2017 exceeded 8.3 billion tons [1]. Nearly half of this plastic was used for disposable packaging, leading to immense generation of plastic waste. By 2050, global plastic waste is estimated to reach 12 billion tons [2]. The limited recycling and reuse of plastics result in the continuous accumulation of plastic waste in the environment [3]. Over time, plastic debris undergoes physical, chemical, and biological processes, fragmenting into micrometer-sized (<5 mm) (called microplastics) and nanometer-sized (≤1 µm) (called nanoplastics) particles [4]. These particles, together with industrially produced plastic particles that are released into the environment, are the major sources of microplastics. In 2004, Richard C. Thompson first introduced the concept of microplastics, which has since garnered increasing research interest and global attention [5]. Based on their origin, microplastics are categorized as primary or secondary microplastics. Primary microplastics are intentionally produced plastic particles with diameters smaller than 5 mm, and they are directly incorporated into products such as cosmetics, exfoliants, and toothpaste; secondary microplastics form via the degradation of environmental plastic [6]. The predominant types of environmental microplastics include polystyrene, polyethylene, polyvinyl chloride, and polypropylene. Notably, polystyrene microplastics, which are frequently detected in the environment, have become the primary focus of microplastic research.

Micro- and nanoplastics are pervasive in the environment, with numerous studies reporting their presence in water, soil, and air [7,8,9,10,11,12]. They have even been detected in remote areas, such as in the deep sea and the Arctic. Research on Arctic ice has revealed approximately 38–234 microplastic particles per cubic meter of ice [13]. Micro- and nanoplastics have also been detected in everyday items, including cosmetics, exfoliants, and toothpaste. Moreover, micro- and nanoplastics can enter organisms through various pathways, such as through the food chain via commercial fish, as well as through canned food and bottled water [14,15,16]. Micro- and nanoplastics have been detected in human feces, placentas, lung tissue, and blood, confirming their ability to enter the human body through multiple routes and thus underscoring the importance of addressing their potential hazards [17,18,19,20].

Current evidence suggests that micro- and nanoplastics are not easily excreted from organisms once ingested, leading to accumulation in organs and tissues [21]. Micro- and nanoplastics have been found to accumulate in the pancreas and gallbladder of zebrafish, as well as in the intestines, liver, and kidneys of mice [22,23,24]. Micro- and nanoplastic accumulation would have potentially toxic effects. For instance, microplastics have been shown to decrease catalase activity in zebrafish and to disrupt energy homeostasis in mice [24,25]. Micro- and nanoplastic-associated adverse effects have been observed in a range of organisms; however, the concentration of micro- and nanoplastics utilized in experimental research far exceeds that found in the environment; therefore, the toxic effects of micro- and nanoplastics at environmental concentration levels remain unclear. Furthermore, research on the adverse effects of micro- and nanoplastics on mammals is limited; thus, definitive conclusions regarding micro- and nanoplastic toxicity cannot be drawn based on existing studies. Additionally, the gut microbiota plays a critical role in digestion and absorption, vitamin synthesis, immune response, and gut barrier function, and it is closely related to host health. Upon entering the intestines, micro- and nanoplastics first interact with the gut microbiota, potentially affecting its composition and function [26]. Consequently, the impact of micro- and nanoplastics on the gut microbiota after entering organisms has become a focus of research.

Academic reviews of micro- and nanoplastics have focused primarily on the presence of micro- and nanoplastics in the environment, the biological exposure pathways, and toxic hazards caused to aquatic organisms, and a few articles have focused on the effects of micro- and nanoplastics on mammals. However, neither the history of research on the detection of micro- and nanoplastics that accumulate in tissues and organs nor the relationship between micro- and nanoplastics and populations has been reviewed in the literature, so the threat posed by micro- and nanoplastics to humans has not been highlighted. In addition, there does not appear to be a scientific summary of the alteration of the metabolites of the gut microbiota and metabolic pathways of the organism caused by micro- and nanoplastics. Furthermore, it is known that micro- and nanoplastics can cause various toxic reactions and affect the function of an organism when they enter the organism. Therefore, this article also summarizes the measures to prevent and alleviate the toxic reactions caused by micro- and nanoplastics after entering an organism. In this article, we review the history of micro- and nanoplastic discovery, the potential pathways through which micro- and nanoplastics enter the human body, ecological toxicity, the toxic effects on marine invertebrates and vertebrates and in mouse models, and alterations in the gut microbiota that are caused by micro- and nanoplastics. Additionally, mitigation of the toxic hazards posed by micro- and nanoplastics through bioactive food components and microbiota transplantation is discussed. The objective of this review is to systematically explore the toxicity of micro- and nanoplastics, raise public awareness of their potential hazards, and provide a reference for future micro- and nanoplastic research. Furthermore, this review is intended to offer data support and a scientific basis for the management of micro- and nanoplastic pollution and its associated environmental policies.

## 2. The History of Micro- and Nanoplastic Discovery

**Figure 1:**
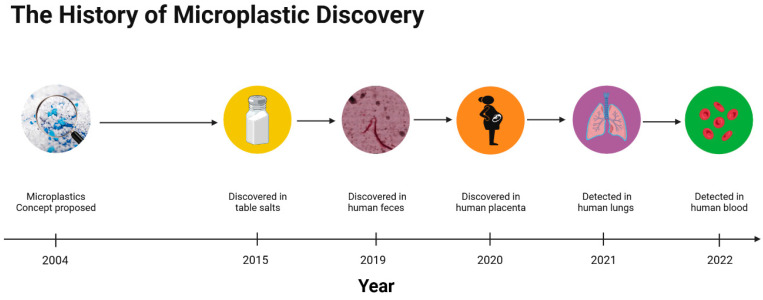
Advances in microplastic discovery. In recent years, scientists have gradually detected microplastics in human tissues.

Since microplastics were first introduced by Thompson in 2004 [5], microplastics have received increasing scientific attention worldwide. Furthermore, in recent years, many scientists have also been concerned about the presence of smaller plastic particles (nanoplastics) in the environment and their environmental and biological toxicity. However, current studies on micro- and nanoplastics are mainly focused on the toxicity of micro- and nanoplastics to marine invertebrates and vertebrates. Thus, there is a lack of studies on mammals and humans, even though scientists have recently found microplastics in human tissues. Therefore, it is of significance to pay attention to the potential toxicities caused by micro- and nanoplastics, and this is also why we need to discuss if micro- and nanoplastics are toxic and hence proposed this review. Furthermore, to our knowledge, this is the first review of the history of microplastics discovery (Figure 1).

In 2015, Yang et al. speculated that microplastics might be present in table salt due to its close contact with seawater and lake water [7]. Yang’s team proceeded to analyze microplastics in salt samples and found that there were 550–681 microplastic particles/kg in sea salt, 43–364 microplastic particles/kg in lake salt, and 7–204 microplastic particles/kg in rock/well salt. Because table salt is prevalent in the human diet, microplastics may enter the body and threaten human health.

In a prospective cohort study in 2019, 8 healthy adult volunteers, aged 33–65 years, from different countries were invited to provide fecal samples without any interventions [17]. When the fecal samples were examined, researchers found that 3–7 types of microplastic, with an average of 20 pieces of microplastic per 10 g, were detected in each fecal sample, which demonstrates that microplastics can indeed enter the human body through oral exposure.

In 2020, scientists began to find microplastics in human tissue. Moreover, for the first time, Ragusa et al. detected microplastics in human placenta [18]. Ragusa recruited six healthy pregnant women to assess the levels of microplastics in the placenta using Raman Microspectroscopy analysis and found twelve 5–10 μm spherical and irregularly shaped microplastic fragments in four placenta samples, including five on the fetal side, four on the maternal side, and three on the chorionic villi. This suggests that microplastics may potentially be harmful to humans across generations. In 2021, Amato-Lourenço analyzed the lung tissue of 20 nonsmoking adults (lung tissue samples were collected after death via a routine autopsy to verify the cause of death) [19]; in 13 of these samples, microplastic particles and fibers were detected, all of which were less than 5.5 μm in size and constituted an average of approximately 0.56 microplastic particles per gram of lung tissue. In 2022, Leslie developed a double-shot pyrolysis–gas chromatography/mass spectrometry method and applied it to determine the level of microplastic content in the whole blood of humans [20]. The investigators measured the microplastic levels in the whole blood of 22 healthy adult volunteers and found that the mean quantifiable total microplastic concentration in the blood was 1.6 μg/mL. These data indicate that at least some of the microplastics absorbed into the body are bioavailable. However, the manner of entry for the microplastics into the blood and the cells involved remains to be studied.

The abovementioned studies show that microplastics are not only ubiquitous in the environment but also detected in humans. As such, the problem of microplastic pollution is not only an environmental issue but also a global public health issue.

## 3. Micro- and Nanoplastic Exposure Routes

**Figure 2:**
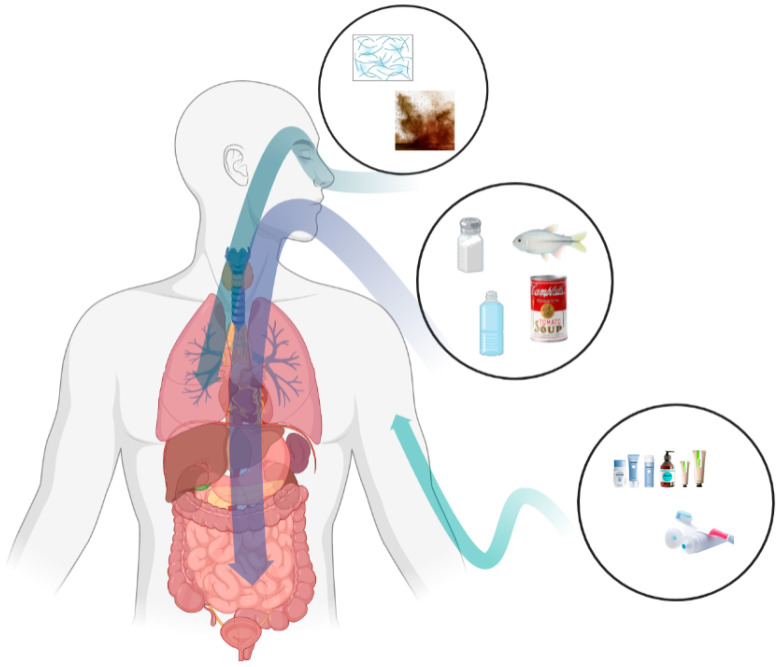
Pathways of human exposure to microplastics mainly include ingestion, inhalation, and dermal contact. Ingestion is the most important route of exposure, and dermal contact is considered the least important route of exposure. For example, micro- and nanoplastics in salt, commercial fish, bottled water, and canned food can enter the human body through ingestion, those in floating fibers and dust in the air can enter the human body through inhalation, and those in toothpaste and skin-cleaning products can enter the human body through skin contact.

The non-degradable and ubiquitous nature of micro- and nanoplastics makes it inevitable that organisms, especially humans, are exposed to microplastics in the environment. Studies have shown that humans are exposed to environmental micro- and nanoplastics mainly through routes such as the ingestion of micro- and nanoplastics through food and food packaging materials, the inhalation of microplastic particles and fibers floating in the air, and daily skin contact with micro- and nanoplastics in cosmetics and skin-cleansing products (Figure 2).

### 3.1. Oral Exposure

Oral exposure is considered to be the predominant microplastic exposure route [27]. Researchers have found micro- and nanoplastics in honey, sugar, bottled water, canned foods, edible salt, and commercial fish [7,14,15,16,28]. Particulate matter enters organisms orally and reaches the gastrointestinal tract, where it may cause inflammatory responses, oxidative stress, alterations in intestinal permeability, and changes in the composition and function of the gut microbiota [26].

First, micro- and nanoplastics in surface water, groundwater, tap water, and bottled water have gradually been detected by scientists. Surface water, the main source of drinking water, has been found to contain high levels of microplastics, and the maximum concentration of microplastics could reach 44,435 items/km^2^ [8]. In addition, Mintenig et al. analyzed the presence of microplastics in groundwater and groundwater-purified drinking water by collecting samples from different locations in the drinking water supply chain; they found that the concentration of microplastics ranged from 0 to 7 particles/m^3^, and the particle size was between 50 and 150 μm [29]. In general, however, the level of microplastic contamination in tap water is low and the concentration of microplastics entering human body is negligible. Therefore, bottled water in plastic packaging has received greater attention. Mason tested bottled water from 9 countries and found an average concentration of 10.4 microplastic particles/L in 259 selected bottles with particle sizes that were greater than 100 μm; the average concentration in the particle size range 6.5–100 μm was 325 microplastic particles/L [16]. In addition, the researchers found that these microplastics matched the common plastics used to make bottle caps, so the contamination may have come from the bottling process and the packaging itself. Schymanski also found microplastics in bottled water, where the concentration in recyclable bottles was 118 ± 88 microplastic particles/L, and in single-use bottles, it was 14 ± 14 microplastic particles/L [30].

It is well known that microplastics are transferred through the food chain into higher-trophic-level organisms. Mattsson’s study found the transportation of microplastics in the algae–daphnia–freshwater fish food chain [14]. Ultimately, microplastics may enter the human body through the food chain. For example, Cauwenberghe used mussels as a vehicle to study the potential threats of microplastics in marine products to humans through the food chain [31]. It was found that when consuming an average serving (e.g., 250 g wet weight) of mussels, a person can consume roughly 90 microplastic particles. Based on this, it can be calculated that the largest consumer of mollusks in Europe will consume up to 11,000 microplastic particles per year. Moreover, as microplastics accumulate, they are biomagnified in higher trophic levels of organisms. Similar to studies on bottled water, those on canned foods have also concluded that incorrect handling during processing can increase the concentration of microplastics in foods [15]. In such a case, Li et al. compared live mussels with pre-treated (frozen or further processed) mussels and found that live mussels contained 0.9 items/g of microplastics, while processed mussels contained 1.4 items/g [32].

Food packaging materials are also the source of microplastics in terms of food pollution. Substances such as the compound monomers and additives left in food packaging materials may migrate into the food with which they come into contact [33]. To extend the shelf life and freshness of food, and to improve the properties of packaging materials, nanoparticles are increasingly being used in food packaging and can also migrate into food [34,35]. Scientists speculate that microplastics in packaging materials may also contaminate food products that come into contact with them. However, there is a relative lack of research on contamination due to microplastic particles in packaging materials and the threat these microplastics pose to the environment and human health.

### 3.2. Respiratory Exposure

Micro- and nanoplastics that are suspended in the air are mainly from synthetic fibers, material wear, and the resuspension of surface microplastics [36]. It is well known that the annual production and use of synthetic fibers have been increasing year-by-year in recent years [37]. Browne’s sampling of household washing machines found that 1 load of laundry can produce > 1900 fibers per wash [38]. In addition, tire wear contributes significantly to the flow of micro- and nanoplastics into the air. The per capita emissions of particulate matter from car tire wear range from 0.23 to 4.7 kg/year [39]. It is estimated that, in the Netherlands, roughly 17,000 t of tire micro- and nanoparticles are created and released into the environment each year [40]. Micro- and nanoplastics enter the air and are deposited together with dust on the surfaces of roads or objects. Low-density polymers are easily resuspended in the air due to the wind or air movement caused by vehicles, and they can enter the human body through the respiratory system.

In addition, researchers have demonstrated that the human body is exposed to micro- and nanoplastics through the respiratory system by measuring microplastic levels in air and human lung tissue, simulating human exposure to microplastics in the air. Liao selected 13 sites by which to sample both indoor and outdoor air and found that the concentration of microplastics in indoor air (1583 ± 1180 n/m^3^) was significantly higher than that in outdoor air (189 ± 85 n/m^3^) [41]. Europeans spend around 90% of their time indoors for work and living, resulting in the majority of human exposure to airborne microplastics occurring indoors [42]. Vianello et al. mimicked human exposure to microplastics in indoor air via a breathing thermal manikin [43]. Their sample analysis showed that all the samples were contaminated by microplastics, and the concentrations ranged from 1.7 to 16.2 microplastic particles/m^3^. More interestingly, scientists have found microplastics in human lung tissue, whereby 33 microplastic particles and 4 fibers were detected in 13 of 20 samples, as well as 39 particles in 11 of the 13 samples, with an average concentration of 0.69 ± 0.84 microplastic per gram of lung tissue, thereby revealing the respiratory exposure pathway to microplastics in humans [19,44].

### 3.3. Dermal Exposure

Skin contact is considered to be the least important but most common exposure route due to the use of microplastics in personal care products (e.g., cosmetics, toothpaste, skin-cleansing products). For purposes such as exfoliation, viscosity adjustment, and emulsification, microplastics are widely used as additives in cosmetic and skin-cleansing products [45]. Depending on the functions, different types and sizes of micro- and nanoplastics are selected. Sun et al. state that the particle size of microplastics that are used in cosmetics ranges from 24 μm to 2 mm, and more than 95% were found to be smaller than 350 μm [46]. Praveena surveyed 214 volunteers from Malaysia on cosmetic and personal care products and found that among the selected products, the particle size of microplastics in face wash/scrubs ranged from 10 to 178 μm [47]. Hernandez et al. drew the same conclusion when they examined three commercial face washes containing polyethylene microbeads, whereby they found nanoplastics with particle sizes that ranged from 24 ± 6 nm to 52 ± 14 nm [48].

There is no study to prove that nanoplastics can cross the skin barrier and enter the organism. However, many studies have shown that when humans are exposed to nanoparticles through the dermal contact route, the nanoparticles can enter the body through the skin barrier and cause toxic reactions [49]. Therefore, scientists have speculated that the direct contact between nanoplastics and human skin during the use of cosmetics and personal care products allows nanoplastics to enter the human body through the skin barrier.

## 4. Toxic Effects of Micro- and Nanoplastics

In recent years, more attention has been paid to the underlying toxic effects of micro- and nanoplastics on the environment and organisms. Studies have revealed that micro- and nanoplastics interacting with the environment can cause eco-toxicity. Micro- and nanoplastics are absorbed by environmental organisms and can enter into consumers through food chain transfer and nutrient transfer, thus causing cytotoxic reactions such as oxidative stress and inflammatory reactions. The normal function of the nervous system and immune system can also be affected by micro- and nanoplastics, causing neurodegenerative diseases and immune system dysfunction.

### 4.1. Eco-Toxicity

Micro- and nanoplastics accumulate when they enter the environment, where they first cause eco-toxicity in environmental organisms such as plants, earthworms, and oysters (Table S1).

Micro- and nanoplastics act on microorganisms in the environment firstly, affecting their activity, metabolism and ability to break down organic matter, thus affecting ecosystem function. Machado et al. focused on the effects of microplastics on the soil microbiota and illustrated that the increase in microplastic concentration drove an improvement in microbiota activity [50]. The same conclusion was obtained in Liu’s study, where high concentrations of polypropylene accelerated the degradation of organic matter in the soil, leading to different metabolite distributions after 7 and 30 d of exposure [10]. However, Lopez-Rojo, when studying the effects of different concentrations of polystyrene microplastics on the decomposition process of dead leaves, demonstrated that the decomposition of leaf litter gradually decreased with increasing microplastic concentrations. There was a significant trend only with the co-existence of harmful substances, and microbiota mediated decomposition as well [51]. Another study suggested that the presence of microplastics in water may affect water ecosystem function, and there is a positive correlation between the concentration of microplastics and the hazards they pose [52]. Additionally, Wang found that microplastic residues caused by the use of mulch in agricultural production contributed to a significant decrease in microbial C, N, and enzyme activities, as well as a significant decrease in microbiota diversity in soil [53].

In addition to affecting the composition of microbiota and their activity, microplastics that are present in the environment are absorbed by plants, affecting seed germination and plant growth. Bosker used a 72 h bioassay to study the effects of microplastic particles of different sizes on cress (*Lepidium sativum*) growth [54]. It was found that, after 8 h of exposure, the germination rate of all microplastic-treated seeds was significantly reduced with the increase in microplastic particle size. In that study, the germination rate of the cress in the 4800 nm nanoplastic group decreased from 78% to 1.7% when compared to the control group. However, more interestingly, the root growth significantly increased after 24 h of exposure to 50 nm nanoplastic, but this decreased significantly after 24 h of exposure to 500 nm nanoplastic. Furthermore, Nolte’s and Zhang’s studies both elucidated that micro- and nanoplastic particles can reduce photosynthetic efficiency by directly altering the chloroplast fatty acid content, as well as changing the structure of the photosynthetic complex and reducing the chlorophyll content of microalgae (*Skeletonema costatum*), thus resulting in the growth inhibition of microalgae [55,56]. Similarly, Green’s study on the effects of microplastics on freshwater ecosystems showed that microplastics significantly reduce the root length and biomass of floating duckweed [52].

Invertebrates can also respond to the eco-toxicity of micro- and nanoplastics. Earthworms (*E. Florida*) were used to study the toxic response to microplastics, and it was concluded that the growth rate of earthworms decreased while the mortality rate increased in the group exposed to high concentrations of microplastics; increasing glutathione (GSH) levels were observed in the earthworms in a dose- and exposure-time-dependent manner [57]. The same conclusion was obtained in Jiang’s experiments, i.e., that nanoplastic exposure may cause oxidative stress, a significant increase in GSH content, and a decrease in superoxide dismutase (SOD) activity in earthworms. In addition, the oxidative damage and DNA damage caused by 14 d exposure to 1300 nm nanoplastics was significantly higher than those for 100 nm nanoplastic [58]. Tlili found that microplastic exposure significantly inhibited the acetylcholinesterase (AChE) activity of wedge clams (*Donax trunculus*), suggesting that the accumulation of microplastics was related to the potential neurotoxicity of wedge clams [59]. Sussarellu’s study showed that microplastic exposure can cause reproductive toxicity via reducing the oocyte diameter and sperm velocity in oysters after 2 months of exposure to polystyrene microplastics [60]. Larval production and the development of offspring in the exposure group were reduced by 41% and 18%, respectively, when compared to the control group. These data suggest that microplastics not only produce toxic responses in the parents but also present transgenerational hazards that affect the growth and development of offspring.

### 4.2. Marine Invertebrates and Vertebrates

Micro- and nanoplastics are absorbed by plants and then pass through the food chain. Then, they are transferred via nutrients into animals, thus causing toxic reactions. Some fish and birds are a better basis for speculating on the harmful effects of micro- and nanoplastics on humans than ecosystems. Therefore, the effects of micro- and nanoplastics on marine invertebrates and vertebrates should be of wide concern. Although an increasing number of scientists are concerned about the toxicity of micro- and nanoplastics, the specific mechanisms of toxicity remain unclear (Table S2).

After entering animals’ bodies through various routes, micro- and nanoplastics are not simply excreted from the body but accumulate in the tissues. In zebrafish embryos, nanoplastics were found in the yolk sac of fertilized eggs 12 h after fertilization, following their exposure to polystyrene nanoplastics 6 h after fertilization. Furthermore, nanoplastics migrate and accumulate in the gastrointestinal tract, liver, pancreas, bile, heart, and even brain of zebrafish during their growth and development, and they also exist in the maternal–infant transmission process [21,61]. Microplastics have been found in terrestrial animals, and seabirds are thought to be the mediators of these pollutants, i.e., they provide a transmission route from the marine environment to the terrestrial environment [62]. Provencher examined 186 thick-billed murres from the eastern Canadian Arctic and found that 11% of thick-billed murres had microplastics in their gastrointestinal tracts with an average of 0.2 ± 0.8 microplastic particles per bird [63].

The accumulation of micro- and nanoplastics in the tissues of organisms first causes histopathological changes. Xia et al. studied the effect of polyvinyl chloride microplastics on the growth of carp and found that, when compared with the normal radiolucent arrangement of carp hepatocytes in the control group, the hepatocytes in the exposure group were visibly loosened and the cell vacuolation was increased [64]. Moreover, micro- and nanoplastics can cause dilated hepatic sinusoids and hypertrophy and necrosis of hepatocytes, and they can increase the extracellular matrix in liver tissues [23,65]. Additionally, exposed to polystyrene microplastics, the zebrafish intestine showed not only thinning of the intestinal wall and congestive inflammation but also impairments and ruptures in the villi and epithelium, lysis of enterocytes, and a disruption of the integrity of the epithelial barrier at high levels [66,67]. In marine medaka (*Oryzias melastigma*), Wang found that microplastics caused damage to gills and testes. Aside from the liver and intestinal tissues, the gills underwent physical changes, such as a loss of gill lamellae and the loosening of gill filaments after 60 days of microplastic exposure. In addition, the testes of marine medaka showed blurring of the spermatophore structure and lysis of the basement membrane when exposed to high concentrations of microplastics [65].

Micro- and nanoplastics generally enter into the organism and cause cytotoxicity due to oxidative stress and inflammatory responses [36]. In a zebrafish study, catalase (CAT) and SOD activities were significantly higher when compared to the control group. In addition, the expression of the inflammatory gene IL-1β and oxidative stress-associated genes was also higher, thus indicating the occurrence of oxidative stress [23,25,64,66,68,69]. It has also been discovered that exposure to micro- and nanoplastics disrupts the feeding behavior of organisms, thus reducing energy intake by affecting the nervous system (i.e., increased feeding times, decreased feeding behavior, etc.) [70,71]. One study showed that microplastics contribute to changes in neurotransmitter activity; specifically, AChE activity was decreased in Amazonian cichlid heads, thus inhibiting their cholinergic neurotransmission [71]. Further, micro- and nanoplastics have been shown to disrupt immune system function, causing the degranulation of primary neutrophil granules and the release of neutrophil extracellular traps (NETs), thus suggesting impaired innate immune function [72]. Similarly, Karami’s study found that microplastics cause decreased globulin levels in fish, which indicates an immunosuppressive response [73].

### 4.3. Mouse Models

Although rarer in these studies, terrestrial mammals are more representative of humans for speculating on the harm of micro- and nanoplastics to humans. Most of the current studies on the effects of micro- and nanoplastics on mammals use mouse models, and the toxicities induced by exposure to micro- and nanoplastics are listed in Table 1.

Similar to those in marine invertebrates and vertebrates, micro- and nanoplastics first give rise to accumulation in the tissues such as liver, kidney, and gastrointestinal tract tissue, and then, they lead to toxic reactions [24,74]. The long-term interaction of micro- and nanoplastics with tissues will result in histopathological changes. For instance, in some studies, the liver index (liver weight/body weight) of the mice in the microplastic-exposed group increased when compared to the control group, and the microplastics induced severe vacuolar degeneration of the liver tissue and caused hepatocyte edema [75,76]. In mice, Jin and Lu also found that micro- and nanoplastics cause a decrease in the amount of intestinal mucin and mucus secretion, as well as inducing intestinal barrier dysfunction [26,77].

Micro- and nanoplastics frequently cause inflammatory responses and oxidative stress when entering organisms. In an experiment investigating the effect of polyethylene microplastics on the development of inflammation in mice, Li demonstrated that the serum levels of IL-1α were significantly higher in the microplastic-exposed group at different concentrations [78]. Additionally, the small intestine of mice fed with high concentrations of nanoplastics exhibited a pronounced inflammatory response with increased expressions of TLR4, AP-1, and RF526. Similarly, Zhao found that interferon-γ, TNF-α, IL-1β, IL-6, and IL-33 mRNA expressions were up-regulated and IL-4, IL-5, IL-10, IL-18, and transforming growth factor-β1 expressions were down-regulated in liver non-parenchymal cells after nanoplastic exposure, indicating that nanoplastics disrupt the inflammatory process in liver tissues [75]. The increased level of oxidative stress that was induced by micro- and nanoplastics in mice was reflected by the decreased activities of antioxidant enzymes containing T-SOD, CAT, and GSH, as well as by the increased reactive oxygen species (ROS) levels [24,76,84]. In addition to the aforementioned, the ROS levels and inflammatory factors IL-6 and TNF-α were reproducible when human-derived cells were exposed to micro- and nanoplastics [79,80].

**Figure 3:**
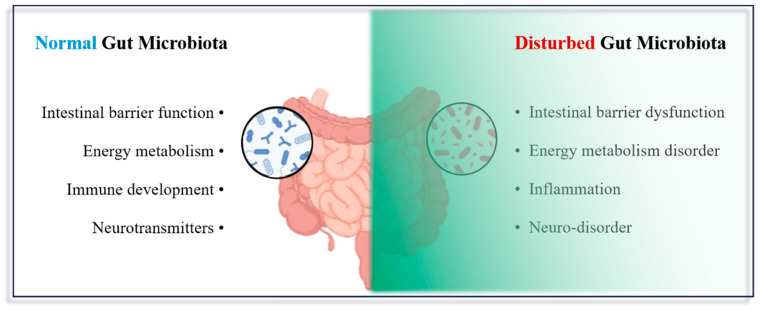
Normal gut microbiota versus micro- and nanoplastic-disturbed gut microbiota.

Interestingly, micro- and nanoplastic accumulation in tissues also causes metabolic disorders in mice. It was found that the continuous accumulation of microplastics in tissues led to a significant decrease in T-CHO and TG, which also led to lipid droplet accumulation and the relative mRNA levels of the key genes related to adipogenesis and triglyceride synthesis being reduced in liver and epididymal lipids, thus causing hepatic lipid metabolism disorders [24,74,81]. The energy metabolism of mice is also disturbed by microplastics, causing lower ATP levels, higher LDH activity, and alterations in energy-related metabolites including creatine, 2-oxoglutarate, and citric acid [24]. In the nervous system, researchers observed a decrease in AChE activity and cholinergic neurotransmission efficiency in mice treated with nanoplastics. In addition, some of these mice showed increased levels of neurotransmitters such as threonine, aspartate, and taurine, suggesting that nanoplastics can induce neurotoxicity in mice. This could also be reflected by nanoplastic-induced behaviors that are similar to anxiety disorders and anti-predator defense responses that occur when mice are confronted with potential predators in field experiments [82]. Furthermore, micro- and nanoplastic exposure induces DNA damage and intergenerational effects in mouse experiments [82,83] (Figure 3).

### 4.4. Impact of Micro- and Nanoplastic Exposure on the Gut Microbiota

The gastrointestinal tract is a particularly complex system that plays an important role in the intake and absorption of nutrients in humans. Micro- and nanoplastics enter and accumulate in the intestine and interact with tissues, affecting the intestinal barrier’s functioning [85]. The intestinal barrier can be divided into the physical barrier, chemical barrier, immune barrier, and microbiological barrier, which consists of a microbiota that maintains intercellular connections and promotes epithelial cell damage repair [86,87]. In their review, Ley et al. noted that there are approximately 10^14^ microbiota present in the human gut, and they emphasized the important role that microbiota play in regard to human health [88]. It has been demonstrated that the host’s health is significantly influenced by the gut microbiota [89]. The gut microbiota is involved in the regulation of host physiological functions, such as the regulation of intestinal motility and secretion, the decomposition of macromolecular polysaccharide compounds in food, participation in the biosynthesis of vitamins and nutrients, participation in the digestion and absorption of nutrients, maintenance of the integrity of the epithelial barrier, and promotion and maintenance of the normal development of the immune system and its activities [90,91,92]. Nevertheless, the accumulation of micro- and nanoplastic particles in the intestine can result in dysbiosis of the gut microbiota, which is closely linked to several illnesses, including diabetes, cardiovascular diseases, hypertension, and others [90,91,92].

#### 4.4.1. Changes at the Compositional Level

Many studies have found that the entry of micro- and nanoplastics into the intestine causes disturbances in the gut bacteria, in terms of both composition and function (Table 2).

Significant changes in the diversity and composition of the gut microbiota are present. In Chinese mitten crab (*Eriocheir Sinensis*), after exposure to 40 mg/L of polystyrene microplastics (with a particle size of 5 μm), the relative abundance of *Firmicutes* and *Bacteroidetes* decreased, whereas the relative abundance of *Fusobacteria* and *Proteobacteria* increased [93]. Further, marine medaka (*Oryzias melastigma*) exposed to polystyrene micro- and nanoplastics at 45 μm and 50 nm showed increased α-diversity of the gut microbiota and a decreased relative abundance of *Bacteroidetes* [94]. Similarly, Zhao et al., who studied the effects of microplastics on zebrafish, found that the exposure of zebrafish to 1–4 μm of polyethylene microplastics for 7 days aroused significant changes in the abundance of *Bacteroidetes*, *Firmicutes*, *Proteobacteria*, and *Verrucomicrobia* in the intestine [95]. In addition, at the genus level, the abundance of *Aeromonas*, *Shewanella*, *Microbacterium*, *Nevskia*, and *Methyloversatilis* increased significantly, while the abundance of *Pseudomonas*, *Ralstonia*, and *Stenotrophomonas* decreased significantly. Soil collembolans exposed to 80–250 μm polyvinyl chloride microplastics for 56 days showed a significant increase in gut microbiota diversity, with a significant change in the microbiota and the appearance of a large number of unique OUTs [96]. In a mouse model, the diversity, amount of bacteria, and *Staphylococcus* abundance were increased, while the *Parabacteroides* decreased significantly, in mice exposed to different concentrations of microplastics (6, 15, 60, and 600 μg/d) [78]. Conversely, in another study, it was found that when mice were exposed to 50 μm and 0.5 μm polystyrene microplastics, their gut micro- and nanobiota diversity was significantly reduced; in the 0.5 μm exposure group, 310 OUT intestinal microbiota were changed, and at the gate level, the relative abundance of *Firmicutes* and *α-Protebacteri* in feces was significantly decreased [77]. Similarly, Jin found that 5 μm polystyrene microplastics led to dysbiosis of the intestinal microbiota, as well as significant changes in 15 bacterial species at the genus level in mice [26].

#### 4.4.2. Changes at the Metabolite Level

It is not enough to study the structure and composition of microbiota; the function of the microbiota should also be considered when assessing whether micro- and nanoplastics are affecting the homeostasis of the gut microbiota. It is acknowledged that gut microbiota participate in regulating the body’s metabolism, intestinal motility, intestinal barrier function, and the digestion and absorption of nutrients, as well as maintenance of the normal function of the immune and nervous systems. Meanwhile, the functions that could interfere with the toxicity of microplastics are mainly a result of the metabolic reprogramming of the gut microbiota (Table 3).

Microplastics cause disruptions in the composition and structure of the gut microbiota and consequently in its metabolic functions: carbohydrate metabolism, lipid metabolism, amino acid, energy metabolism, etc. Microplastic-exposure-induced dysbiosis of the gut microbiota has been reported to significantly alter carbohydrate metabolism; the expression of the pentose phosphate metabolic pathway (i.e., a glucose catabolic pathway that is prevalent in microbiota) was significantly reduced [97]. In addition, fructose and mannose metabolism in human Caco-2 cells was inhibited with down-regulated mannose metabolism gene expression. Inhibition of carbohydrate metabolism also occurred in marine medaka (*Oryzias melastigma*) and in mealworms (*Tenebrio molitor*) [98,99]. In addition, normal lipid metabolism was disturbed after long-term exposure to polystyrene microplastics, both in the gut microbiota and in the host [98,100]. In a recent study, microplastics were demonstrated to reduce the level of taurocholic acid (TCA) via increasing the ratio of *Bifidobacteria* to *Bacteroide* in the intestine, thereby reducing lipid absorption in mice [101]. Microplastic exposure also alters the amino acid and energy metabolism of chicken gut microbiota; this is accompanied by a significant decrease in L-serine and ornithine levels, which have a variety of important functions in the developmental stages, such as the synthesis of neurotransmitters, proteins, and sphingolipids [102]. Peng’s study showed that polystyrene microplastics interfere with histidine metabolism in the mealworm gut microbiota, which is followed by alterations in metabolic pathways, such as pentose phosphate metabolism, alanine metabolism, and glutamate metabolism [99]. In another study, in mouse gut microbiota, microplastic exposure for 6 weeks resulted in significant differences in the metabolic pathways of tyrosine-functional genes [26].

Moreover, microplastics affect the synthesis of bile acids by intestinal flora, the production of short-chain fatty acids and neurotransmitters, and the normal function of the body. It is known that Gram-positive bacteria promote the synthesis of bile acids in the intestine. In an experiment on the effect of polyethylene microplastics on the intestinal microbiota, it was found that the proportion of *Firmicutes* decreased, resulting in a reduction in secondary bile acid synthesis [97]. Qiao’s study showed that exposing mouse intestinal microbiota to 5 μm and 70 nm polystyrene micro- and nanoplastics resulted in a decrease in short-chain-fatty-acid-producing bacteria and an increase in Gram-negative bacteria [103]. Because short-chain fatty acids and lipopolysaccharide production by Gram-negative bacteria are closely related to intestinal barrier function, the investigators suggested that micro- and nanoplastics acting on intestinal tissues may indirectly disrupt barrier function by modulating the microbial structure. In addition, decreases in *Lactobacillus* and *Bifidobacterium*, which are the main γ-aminobutyric acid (GABA)-producing bacteria in the gut, result in a decrease in the GABA content, which thus further alters neurotransmitter synthesis and affects the neurotransmission and behavior of fish [104]. Studies on how micro- and nanoplastics affect the function of the immune system through the gut microbiota are incredibly limited. However, Kang et al. found that micro- and nanoplastics affect gut mucus secretion; this was noted when there was a significant increase in the amount of mucus in the exposed group when compared to the control group. Because mucus contains a variety of microbiota, it plays a key role in a variety of physiological functions, including immune function [94].

### 4.5. Micro- and Nanoplastics and Population

Although more and more scientists are studying micro- and nanoplastics, most of the studies on their toxicity involve ecological, non-mammalian, and laboratory mouse models, but relatively few studies have been conducted on human populations. Current research on micro- and nanoplastics relevant to humans is limited to the discovery of their accumulation in human tissues and in vitro experiments with human cells. After scientists discovered the presence of microplastics in placenta by Raman spectroscopy in 2021 [18], in 2023, Zhu et al. used infrared spectroscopy to characterize microplastics in placenta. A total of 11 types were found in the placenta, with particle sizes ranging from 20.34 μm to 307.29 μm, most of which were predominantly fragments (<100 μm) and fibers (200–307.29 μm) [105]. Furthermore, systemic toxicity of microplastics to the placenta was postulated by a machine learning approach [106]. One researcher found that polycarbonate, polyethylene terephthalate, and polystyrene exhibited the highest toxic effects on all enzymes. These microplastics can effectively identify the site of enzyme activity and pose a significant risk to the placenta through inhibition of key enzymes. Whether these micro- and nanoplastics in the placenta make it into the fetus is critical. Braun et al. collected maternal placenta and meconium to test for the presence of microplastics [107]. The results showed that polyethylene, polypropylene, polystyrene, and polyurethane were present in placenta and meconium, and their particle sizes were all > 50 μm. In addition to the placenta, the researchers found through a small prospective cohort study that microplastics in fetuses may also come from breastfeeding, feeding bottles, and plastic toy use [108]. These microplastics accumulate in the fetus, and liu et al. found that they can affect the composition of fetal gut microbiota and cause microbiota disturbance [109]. The gut microbiota was mainly composed of Proteus, Bacillus, and Villus, and the Chao index of gut microbiota and polystyrene was negatively correlated. The concentration of microplastics was positively correlated with *Pseudomonas aeruginosa*, *streptococcus*, and *Clostridium*. In addition, Huang et al. detected the presence of microplastics in almost all sputum samples collected from people with respiratory diseases [110]. In addition, polyurethane was the main microplastic detected, followed by polyester, polyvinyl chloride, etc. According to the data analysis, it was revealed that the exposure of microplastics may be related to smoking or not smoking and invasive air tube inspection. Furthermore, microplastics have been found in human testicles, semen, and intestines [111,112]. In addition, scientists used vitro experiments to study the possible effects of micro- and nanoplastics on humans. Annangi used human nasal epithelial cells to study the possible effects of polystyrene nanoplastics in the air and found that after exposure to nanoplastics, reactive oxygen species increase, mitochondrial membranes lose potential, and autophagy accumulation regulates the autophagy process [113]. Furthermore, human fibroblasts exposed to polystyrene nanoplastics also showed mitochondrial damage and disrupted the expression of caspase3, caspase9, cytochrome c, and other related proteins to induce cell apoptosis [114]. Polystyrene nanoplastics can be internalized by human alveolar basal epithelial cells and human colorectal adenocarcinoma cells [115] and damage the cell vitality of liver cancer cells [116]. All of this indicates the potential threat of micro- and nanoplastics to human health.

In conclusion, not only microplastics but also nanoplastics are found to be widespread. It is well known that the concentration and size of contaminants can significantly affect their toxicity. Scientists have realized the seriousness of the problem and begun conducting in-depth research. First, microplastics’ different particle sizes affect their accumulation in different tissues. Deng et al. used 5 μm and 20 μm polystyrene microplastics to investigate whether different particle sizes would affect their accumulation in tissues and organs. They found that although microplastics of different sizes were accumulated in liver, kidney, and intestine, their tissue accumulation kinetics and distribution were strongly dependent on their particle sizes [24]. After 4 weeks of exposure, the concentrations of 5 μm microplastics in liver, kidney, and intestine reached 4.42 × 10^6^ ± 4.23 × 10^5^ items/g, 1.38 × 10^7^ ± 1.36 × 10^6^ items/g, and 2.03 × 10^7^ ± 2.00 × 10^6^ items/g, respectively. However, the concentrations of 20 μm microplastics were relatively low: 1.73 × 10^5^ ± 1.68 × 10^4^ items/g, 1.78 × 10^5^ ± 1.91 × 10^4^ items/g, and 1.77 × 10^5^ ± 1.86 × 10^4^ items/g. Yang et al. also found that the absorption rate constant of microplastics with smaller particles is larger, while that of larger particles is smaller [74]. Compared to microplastics, nanoplastics have smaller particle sizes and are more likely to enter organisms through various routes. For example, when zebrafish embryos were exposed to 100 nm, 500 nm, and 1 μm micro- and nanoplastics, large amounts of nanoplastics were found to be deposited on the chorionic surface and yolk sac of the embryos in the 100 nm and 500 nm exposed groups, and they even were observed in the brains of larvae [117]. However, in the 1 μm microplastic exposure group, microplastics were only deposited on the surface of the embryonic chorionic membrane and did not enter the embryo interior and larval brain tissue. More interestingly, Zhang et al. studied the accumulation of 70 nm, 200 nm, and 500 nm polystyrene nanoplastics using human alveolar basal epithelial cells (A549) and human colorectal adenocarcinoma cells (Caco-2) [115]. It was found that all 3 sizes of nanoplastics could be internalized, but the numbers of nanoparticles of 200 nm and 500 nm were lower than that of 70 nm nanoplastics. These studies show that smaller micro- and nanoplastics seem to more easily enter organisms and accumulate in tissues, so smaller nanoplastics should deserve more attention. In addition to the differences in bioaccumulation, the scientists also found that smaller plastic particles seem to cause more serious biotoxicity and biohazards. When human hepatoma cells (HepG2 cells) were exposed to 50 nm, 100 nm, 1 μm, and 5 μm micro- and nanoplastics, scientists found that smaller aminated particles (50 nm, 100 nm) were more harmful to cell viability than larger aminated particles (1 μm, 5 μm) [116]. In addition, Wang et al. also found that the smaller the particle size of nanoplastics was, the more easily they were bound to superoxide dismutase (SOD) in cells to form complexes, and the smaller nanoplastics (100 nm) induced more significant changes in SOD activity (20% increase in activity). However, nanoplastics with larger particle size (200 nm and 1 μm) had little effect [118].

Furthermore, exposure concentration is also an important factor affecting the toxicity of micro- and nanoplastics. For example, Banerjee et al. found that not only the particle size but also the concentration was an important factor in their toxicity [116]. The effects of aminated nanoplastics (50 nm and 100 nm) on cell activity increased with the increase in the concentration, and the toxicity was greatest when the maximum exposure concentration was reached (100 μg/mL). Interestingly, however, Hu et al. found that nanoplastics had a double effect on the effects of Pseudomonas aeruginosa (PAO1) [119]. Using 0.1 mg/L, 20 mg/L, and 50 mg/L polystyrene nanoplastics to culture PAO1, the researchers found that 20 mg/L and 50 mg/L polystyrene nanoplastics significantly inhibited the nitrate reduction process, and the expression of related denitrification genes was also significantly down-regulated. However, denitrification of PAO1 was promoted when it was exposed to 0.1 mg/L polystyrene nanoplastics. The double effect was also reflected when Macrobrachium nipponense was exposed to different concentrations of polystyrene nanoplastics [120]. The study found that with the increase in the concentration, the activity of SOD, CAT, and other antioxidant enzymes generally decreased, while the expression of antioxidant-related genes showed a trend of first increasing and then decreasing. The expressions of SOD and CAT genes in the 5 mg/L and 10 mg/L exposure groups were significantly higher than those in the control group, and the expressions of SOD and CAT genes were significantly lower than those in the control group when concentration increased to 20 mg/L and 40 mg/L. In addition, the trend of immunoenzyme activity in Macrobrachium nipponense was consistent with that of antioxidation-related genes. These results suggest that low concentrations of polystyrene micro- and nanoplastics appear to enhance the survival of organisms in the environment, but high exposure has toxic effects.

## 5. Preventive Strategies

Most of the current studies focus on the dangers of plastics to organisms and humans, but the ultimate goal is to understand how to prevent these hazards (Table 4).

Anthocyanin is a safe and non-toxic natural pigment extracted from bayberry, mulberry, strawberry, blueberry, and other berries. It functions as an antioxidant and anti-inflammatory, as well as having anti-apoptosis, anti-cancer, anti-diabetes, and neuroprotective properties [121,122]. Cyanidin-3-glucoside (C3G) is one of the functional factors of anthocyanins, and it has been reported to alleviate the toxic effects of polystyrene by promoting the excretion of polystyrene from feces and reducing the harm of locomotion behavior in terms of head and body bending time [121,122,123,124,125,126]. The mechanisms by which C3G alleviates the toxic reactions caused by polystyrene include the activation of autophagy, the remodeling of intestinal flora, and changing metabolic pathways. Autophagy is necessary for C3G to alleviate polystyrene-induced cytotoxicity. By activating the Sirt1-Foxo1 signaling pathway, C3G increases LC3 levels in cells (an increased expression level can activate autophagy) and decreases p62 levels (a decreased expression level promotes autophagosome formation), which causes autophagy and promotes polystyrene degradation [122]. Through 16S rRNA high-throughput sequencing, Chen found that the intestinal microbiota composition was changed after PS and C3G treatment in mice [123]. As a result, the numbers of *Dubosiella* used as intestinal probiotics decreased after 6 weeks of PS administration, while the decreasing trend was significantly alleviated after C3G intervention. Compared with the polystyrene-exposed group, the level of probiotic genes in the C3G-treated group was significantly increased. C3G was also involved in energy metabolism and improved mitochondrial dysfunction. Studies have found that C3G can correct polystyrene-induced mitochondrial dysfunction and increase the ATP content of cells and nematodes by activating the AMPK/SIRT1/PGC-1α signaling pathway [125]. Moreover, the accumulation of polystyrene in the body will cause oxidative stress, thus producing excessive ROS and superoxide anions (O^2-^). By alleviating the effects of oxidative stress, C3G can delay age-related physiological decline and aging, as well as prolong the lifespan of nematodes [122].

**Table 4:** Approaches used to counteract the toxic effects of microplastic exposure.

Species	Chemicals and Strategies	Prevention Approaches and Effects	Reference
Mouse and Caco2 cells	Polystyrene and C3G	Triggers autophagy by activating the Sirt1-Foxo1-1 signaling pathway to alleviate polystyrene-induced toxicity The co-localization of polystyrene and lysosomes was observed, suggesting that PS is encapsulated and degraded The co-localization of autophagy genes and PS was found, suggesting that autophagy is involved in the beneficial effects of C3G	[122]
Mouse	Polystyrene and C3G	C3G remodels the gut microbiota in mice and affects the gene abundance of bacterial functional pathways Differential metabolic pathways and metabolites were discovered Significantly increased levels of probiotics	[123]
Mouse	Polystyrene and C3G	Effectively reduces tissue accumulation and increases polystyrene excretion from feces C3G regulates intestinal microbiome disturbance and regulates inflammatory function genes Triggered alterations in functional pathways in response to xenobiotic polystyrene, thus reducing bacterial functional genes associated with disease and inflammation	[124]
*C. elegans* and Caco2 cells	Polystyrene and C3G	Recovery of polystyrene-induced ATP reduction, achieved by activating the AMPK/SIRT1/PGC-1α signaling pathway and by improving mitochondrial dysfunction Increased fecal polystyrene efflux	[125]
*C. elegans*	Polystyrene and C3G	C3G can ameliorate polystyrene-induced oxidative stress and shorten its lifespan C3G can significantly enhance the expression of DAF-16 pathway-related genes	[126]
*C. elegans*	Polystyrene and FMT	Promotes intracellular GSH production by activating the PMK-1/SKN-1 pathway and also reduces the production of ROS and O^2-^ induced by polystyrene FMT significantly alleviated the harm caused by the polystyrene-induced inhibition of nematode body lengths and motility behaviors	[127]

Fecal microbiota translocation (FMT) is another effective strategy to deal with polystyrene toxicity. In an experiment regarding microbiota transplantation, it was found that the GSH content in the polystyrene-exposed group was decreased and the consumption of GSH was increased, while the group treated with FMT exhibited accelerated generation of GSH, which thus alleviated oxidative stress by increasing the expression level of GSH synthase [127]. Overall, prevention and treatment strategies for addressing the public health problems caused by microplastics are still limited and there is an urgent need for further exploration.

## 6. Conclusions and Perspectives

Microplastic pollution has pervaded the environment. Human exposure and the cumulative uptake of these microplastics are expected to increase over time, and this phenomenon has aroused wide concern among scientists. A growing number of scientists, however, have studied the potential hazards of microplastics when exposed to ecosystems, invertebrates and vertebrates, and laboratory mouse models. Knowledge about the toxicity of microplastics is still limited.

(i) Most of the current studies on the toxicity of micro- and nanoplastics have focused on the ecological environment and non-mammalian and laboratory mouse models. So far, what we know about micro- and nanoplastics and human health includes the fact that micro- and nanoplastics have accumulated in human tissues and organs, and relatively little research has been done on the harm they cause. However, the accumulation of micro- and nanoplastics in human tissues having not been discovered until recent years, the ethical limitations of collecting human specimens, and our current limited understanding of the toxicity of micro- and nanoplastics and the biomarkers that reflect their toxicity have limited scientists to conducting epidemiological studies. Determining whether micro- and nanoplastics have direct or indirect relationships with the occurrence and development of human diseases still requires scientists to continue efforts and exploration.
(ii) There are limited data on the ecological, biological, and human toxicity of micro- and nanoplastics under environmentally relevant conditions. Exposure concentrations of the microplastics used in the laboratory study were significantly higher than those associated with the environment, so the scientists speculate that the laboratory results may overstate the harm caused by micro- and nanoplastics at the environmentally associated concentrations. In addition, extensive studies are still needed to elucidate the pathological mechanisms by which microplastics cause toxic hazards at the cellular and tissue levels and the health consequences of long-term exposure.
(iii) In addition, factors affecting the toxicological role of microplastics, such as sex differences, the dose–response relationship, exposure frequency, and the type and size of microplastics have not yet been thoroughly investigated. Therefore, it is urgent to conduct more in-depth research on the factors influencing microplastics’ toxicity, microplastics-related knowledge, and potential risks, so as to provide a scientific basis for policy makers to cooperate with each other, solve this pressing environmental problem, and protect human health.

However, we believe that this review may have some limitations. In summary, there is a lack of population studies on micro- and nanoplastics, so although our aim was to study the toxic hazards of micro- and nanoplastics in the population, unfortunately, based on our literature search, there are few data available on toxic effects of microplastics at the population level. The relatively few data on population studies in this review still do not allow for a better assessment of the hazards of micro- and nanoplastics in humans.

## Figures and Tables

**Table 1:** Toxic effects of microplastic exposure on laboratory mice.

Properties of Microplastics Used	Toxicity	Reference
Polystyrene microplastics (5–20 μm)	Accumulated in the kidney, liver, and intestine of mice, and the highest bioaccumulation factor was found in the intestine	[74]
Polystyrene microplastics (5 μm)	Accumulated in the intestinal tissues and also reduced intestinal mucus secretion and impaired intestinal barrier function Changes in the intestinal flora diversity with a significant decrease in actinomycetes Significant changes in metabolic pathways such as pyruvate metabolism, tyrosine metabolism, and fatty acid biosynthesis	[26]
Polystyrene nanoplastics (0.5 μm)	Increased liver weight, liver index, and liver function indicators Up-regulation of interferon-γ, TNF-α, IL-1β, IL-6, and IL-33 mRNA expression in non-parenchymal hepatocytes Down-regulation of IL-4, IL-5, IL-10, IL-18, and transforming growth factor-β1 expression	[75]
Polystyrene microplastics (5 μm)	Accumulated in the liver tissue accompanied by tissue vacuolar degeneration, chronic inflammatory cell infiltration, and hepatocellular edema Decreased T-SOD, CAT, and GSH activities and increased MDA levels L02 hepatocyte rate of apoptosis increased	[76]
Polystyrene micro- and nanoplastics (0.5 μm and 50 μm)	Reduced body weight, liver weight, and lipid weight in the mice Decreased intestinal mucus secretion Relative abundance of *Firmicutes* and *α-Protebacteri* were reduced Lower liver triglyceride and total cholesterol levels Decreased mRNA levels of certain key genes associated with adipogenesis and triglyceride synthesis	[77]
Polystyrene microplastics (10–150 μm)	Increased abundance of *Staphylococcus* and decreased abundance of *Paramecium* Elevated IL-1α levels The small intestine showed a significant inflammatory response, as well as increased expression of TLR4, AP-1, and IRF526	[78]
Polystyrene microplastics (5 μm and 20 μm)	Accumulation in both the kidney and intestine, with tissue accumulation kinetics and distribution patterns dependent on microplastic particle size Caused disturbances in energy and fat metabolism, as well as causing oxidative stress and neurotoxic reactions	[24]
Polyethylene micro- and nanoplastics (3–16 μm, 100 nm, and 600 nm) Polystyrene micro- and nanoplastics (10 μm, 40 nm, and 250 nm)	Increased ROS generation	[79]
Polypropylene microplastics (<200 μm)	Elevated IL-6 and TNF-α levels Elevated ROS levels Caused erythrocyte hemolysis in a concentration-dependent manner Increased the secretion of histamine, which induces allergic reactions at the cellular level	[80]
Polystyrene microplastics (1 μm, 4 μm, and 10 μm)	Accumulation in the intestinal tract Causes a decrease in cell viability at higher concentrations Macrophage uptake of microplastics was followed by polarization	[81]
Polystyrene nanoplastics (23–26 nm)	Accumulation in the mouse brain Changes in anxiety-like behavior and anti-predator defense responses in the face of predators Reduced DPPH radical scavenging activity and reduced total GSH content Appearance of DNA damage	[82]
Polystyrene microplastics (5 μm)	Caused metabolic disorders, intestinal flora dysbiosis, and intestinal barrier dysfunction in the mother Caused intergenerational effects with long-term metabolic consequences in the F1 and F2 mice The possibility of hepatic lipid accumulation in the F1 generation mouse in adulthood	[83]

**Table 2:** Gut microbiota composition changes after microplastic exposure.

Species	Properties of Microplastics Used	Changes in Intestinal Microbiota	Reference
Chinese mitten crab (*Eriocheir sinesis*)	Polystyrene microplastics (5 μm)	Reduced diversity of gut microbiota The relative abundance of *Firmicutes* and *Bacteroidetes* decreased, and the relative abundance of *Fusobacteria* and *Proteobacteria* increased	[93]
Marine medaka (*Oryzias melastigma*)	Polystyrene micro- and nanoplastics (45 μm and 50 nm)	Increased α-diversity of gut microbiota in the exposed group The relative abundance of *Bacteroidetes* and *Vicingus* decreased The relative abundance of *Lewinella*, *Pseudomonas*, *Thalassospira*, and *Parahaliea* increased	[94]
Larval zebrafish	Polystyrene microplastics (1–4 μm)	Firmicutes, Bacteroidetes, Proteobacteria, and Verrucomicrobia changed significantly at the gate level At the genus level, the relative abundance of Aeromonas, Shewanella, Microbacterium, Nevskia, and Methyloversatilis increased significantly, while the relative abundance of Pseudomonas, Ralstonia, and Stenotrophomonas decreased significantly	[95]
Collembolans	Polyvinyl chloride microplastics	Increased gut microbiota diversity The relative abundance of *Bacteroidetes* decreased, and the relative abundance of *Firmicutes* increased	[96]
Mouse	Polyethylene microplastics (10–150 μm)	Increased diversity of gut microbiota The relative abundance of *Staphylococcus* increased, and the relative abundance of *Parabacteroides* decreased	[78]
Mouse	Polystyrene micro- and nanoplastics (50 μm and 0.5 μm)	At the gate level, the relative abundance of *Firmicutes* and *α-Protebacteri* was reduced A total of 6 and 8 bacterial species were altered by exposure to 0.5 and 50 μm micro- and nanoplastics, respectively, at the genus level	[77]
Mouse	Polystyrene microplastics (5 μm)	Changes in α-diversity and β-diversity occurred A total of 15 species were significantly altered at the genus level	[26]

**Table 3:** Gut microbiota changes in metabolites due to microplastic exposure.

Species	Properties of Microplastics Used	Changes in Gut Microbiota Metabolome	Reference
Simulation in vitro with human cell Caco-2 and gut microbiota	Polyethylene microplastics (30–140 μm)	Significantly lower expression in the metabolic pathway of pentose phosphate metabolism Mannose metabolism gene expression is down-regulated, and fructose and mannose metabolism is inhibited Decreased secondary bile acid synthesis	[97]
Marine medaka (*Oryzias melastigma*)	Polystyrene microplastics (2.5 μm)	Significant reduction in carbohydrate metabolic pathways Significant up-regulation of fat metabolic pathways	[98]
Mealworms (*Tenebrio molitor*)	Polystyrene microplastics	Interference with the starch and sucrose metabolism of intestinal bacteria Interference with the histidine metabolism of gut microbiota (related to pentose phosphate metabolism, alanine metabolism, and glutamate metabolism pathways) Disrupts glyoxylate and dicarboxylic acid metabolism (key energy metabolic pathways)	[99]
Mouse	Polystyrene microplastics (5 μm)	Significant alterations in the metabolic pathways of tyrosine functional genes	[26]
Rare minnow (*Gobiocypris rarus*)	Polystyrene microplastics (1 μm)	Altered amino acid metabolic pathways Disorders of normal lipid metabolism	[100]
Mouse	Polystyrene microplastics (5 μm)	Decreased TCA and triglyceride levels	[101]
Chicken	Polyethylene microplastics	Significant alterations in amino acid metabolic pathways (accompanied by significant decreases in L-serine and ornithine levels)	[102]
Mouse	Polystyrene micro- and nanoplastics (5 μm and 70 nm)	Significant damage to the intestine and decreased expression of tight-junction proteins Decrease in short-chain-fatty-acid-producing bacteria and increase in Gram-negative bacteria	[103]
Discus fish (*Symphysodon aequifasciatus*)	Polystyrene nanoplastics (~80 nm)	*Lactobacillus* and *Bifidobacterium* (i.e., the main GABA-producing bacteria) decreased Altered neurotransmitter synthesis that resulted in behavioral changes in discus fish	[104]
Marine medaka (*Oryzias melastigma*)	Polystyrene micro- and nanoplastics (45 μm and 50 nm)	The group exposed to 50 nm polystyrene nanoplastics exhibited stronger oxidative stress The group exposed to 45 μm polystyrene microplastics exhibited intestinal damage, as well as an increase in gut mucus secretion and alterations to the gut microbiota	[94]

## Supplementary Materials

The following supporting information can be downloaded at https://www.mdpi.com/article/10.3390/metabo13060739/s1. Table S1: Toxic effects of microplastic exposure to environmental organisms; Table S2: Toxic effects of microplastic exposure to marine invertebrates and vertebrates.
