# Supplementary material for: Are Microplastics Toxic? A Review from Eco-Toxicity to Effects on the Gut Microbiota
Source: Metabolites. 2023 Jun 9;13(6):739. doi: 10.3390/metabo13060739 (PMC10304106; doi:10.3390/metabo13060739)
Supplement: Supplementary file 1 [file metabolites-13-00739-s001.zip › metabolites-2381487-supplementary.pdf]

Table S1. Toxic effects of microplastic exposure to environmental organisms.

Samples	Properties of MPs/NPs	Toxicity	Reference
Soil		A significant relationship between microplastic concentration and microbial activity<br>The microbiota activity of the polyacrylic and polyester group was significantly lower than that of the control group	[50]
Soil	Polypropylene microplastics (<180μm)	The higher the concentration of microplastics, the higher the nutrient content in the solution of dissolved organic matter<br>Promoting enzyme activity and facilitates the accumulation of dissolved organic C, N, and P	[10]
Mixed leaf litter	Polystyrene microplastics （10μm）	Causes detritivore mortality, but does not affect growth<br>The decomposition of leaf litter decreases with increasing microplastic concentration (more pronounced in the presence of harmful substances)<br>Microbially mediated decomposition decreases with increasing microplastic concentration	[51]
Freshwater ecosystem	microplastics	Significant reduction in root length of duckweed and biomass of Phytoplankton<br>The abundance of New Zealand mud snails (Potamopyrgus antipodarum) increased	[52]
Soil	Plastic film residue	PAE concentrations increased with increasing plastic film residues<br>The soil microbial carbon and nitrogen, enzyme activities, and microbial diversity decreased significantly	[53]
Cress (*Lepidiumsativum*)	Fluoro-Max Green Fluorescent Polymer nanospheres (50、500、4800nm)	The germination rate of cress decreased after 8h of microplastics treatment: from 78% to 1.7% after 4800nm microplastic treatment, and there was no difference in germination rate after 24h of exposure	[54]

		Differences in root growth emerged after 24 h of exposure, and the differences were not significant after 48 and 72 h	
Marine microalgae (Skeletonema costatum)	Polystyrene nanoplastics （110nm）	Alteration of chloroplast fatty acid content affects the structure of photosynthetic complexes in microalgae, thus affecting microalgal growth	[55]
Marine microalgae (*Skeletonema costatum*)	pure polyvinyl chloride micro-plastics(1mm)	It has a significant inhibitory effect on the growth of microalgae, with a maximum inhibition rate of 39.7% after 96 hours of exposure<br>High concentration of mPVC reduces chlorophyll content and photosynthetic efficiency of algae	[56]
Earthworm (*Eisenia foetida*)	Polypropylene microplastics (<150μm）	Accumulation in earthworm<br>Decreased growth rate and increased mortality<br>Increased GSH levels in the body in a dose-dependent manner, with an increasing trend of GSH levels over time	[57]
Earthworm (*Eisenia fetida*)	Polystyrene nanoplastics（100nm and 1300nm）	Accumulation in intestinal tissues and damage to intestinal cells<br>Significantly altered GSH and SOD levels<br>Caused DNA damage	[58]
Wedge clam (*Donax trunculus*)	Polyethylene and polypropylene nanoplastics（100nm-400nm）	Gills are the first target organ for microplastic accumulation<br>Significant inhibition of total AChE activity in gills and digestive glands<br>Caused oxidative stress	[59]
Oyster	Polystyrene microplastics（2μm and 6μm）	Significantly higher consumption and uptake efficiency of microalgae in the exposed group and disturbed energy metabolism<br>Significantly lower oocyte number, diameter, and sperm velocity in the exposed group<br>Decreased larval production and development in the progeny of parents in the exposed group	[60]

Table S2. Toxic effects of microplastic exposure to marine invertebrates and vertebrates.

Samples	Properties of microplastics used	Toxicity	Reference
Zebrafish (*Danio rerio*)	Polystyrene nanoplastics (average diameter of 51 nm)	● Accumulated in the yolk sac 24 h after fertilization and migrates to the gastrointestinal tract, bile, liver, pancreas, heart, and brain during the whole development process<br>● Decreased accumulation in all organs during purification<br>● Did not result in significant death, malformation, or changes in mitochondrial bioenergetics, but decreased heart rate<br>● Altered larval behavior, as evidenced by insufficient swimming activity	**[21]**
Zebrafish (*Dani rerio*)	Polystyrene nanoplastics （42nm）	● Significantly reduced GSH reductase activity in F0 generation brain, muscle, and testis, but did not affect mitochondrial functional parameters in heart and gonads<br>● Polystyrene accumulates in the yolk sac, gastrointestinal tract, liver, and pancreas in the F1 generation, and bradycardia occurs in the F1 generation<br>● Reduced GSH peroxidase activity and thiol levels in the F1 generation	[61]
Seabirds		● The presence of anthropogenic particles in fecal precursors of both Fulmarus glacalis and Uria lomvia<br>● It is estimated that fulmars and murres deposit 3.3-45.5 million artificial particles into the environment respectively during the breeding period	[62]
Thick-billed murre (*Uria lomvia*)		● About 11% of the birds had at least one piece of plastic debris in their gastrointestinal tract	[63]

Carp (*Cyprinus carpio*)	polyvinyl chloride microplastics	• Significantly inhibited the growth and weight gain of juvenile fish<br>• GSH peroxidase activity increases and then decreases with increasing exposure concentration<br>• A negative correlation between SOD and catalase activities<br>• Significant reduction in malondialdehyde levels<br>• Transcript levels of cytochrome P450 1A and GSH Stransferase increase and then decrease in the liver<br>• Cytoplasmic vacuolation in the liver	[64]
Zebrafish (*Danio rerio*)	Polystyrene micro- and nanoplastics (5μm, 20μm, 70nm)	• After 7 d of exposure, microplastics with a diameter of 5 μm accumulated in the gill, liver, and intestine, and 20 μm microplastics accumulated only in the gill and intestine of the fish<br>• Causes liver inflammation and lipid accumulation<br>• Significantly elevated SOD and catalase activities, caused oxidative stress<br>• Changes in the metabolic profile of fish liver and disruption of fat and energy metabolism	[23]
Marine medaka (*Oryzias melastigma*)	Polystyrene microplastics (10μm)	• Accumulation of microplastics in gills, intestine, and liver, leading to oxidative stress and histological changes<br>• Eased gonadal maturation in females and reduces female fecundity<br>• Significant negative regulation of hypothalamic-pituitary-gonad in female fish<br>• Delayed hatching time of offspring, reduced hatching rate, heart rate, and body length of offspring	[65]
Zebrafish	Polystyrene microplastics（5μm）	• Inflammatory response and oxidative stress in the intestine<br>• Significant changes in the gut microbiome and tissue metabolic characteristics, mostly associated with oxidative stress,	[66]

		inflammation, and lipid metabolism	
Zebrafish （*Danio rerio*） and nematode (*Caenorhabditis elegans*)	PA, PE, PP, PVC and PS (70μm)	● Microplastic exposure for 10 d, no or low lethality of D. rerio<br>● Caused intestinal damage, including villi rupture and intestinal cell division<br>● Exposure to 2d significantly inhibited nematode survival, body length, and fecundity<br>● Decreasing calcium levels in the intestine and increasing GSH Stransferase expression	[67]
Larval zebrafish (*Danio rerio*)	Polystyrene nanoplastics (468nm and 508nm)	● After fertilization 4h begins exposure and first attaches to the embryonic chorion and then enters the larval gastrointestinal tract<br>● Reduced swimming speed and swimming distance<br>● Upregulation of inflammation and oxidative stress-related gene expression	[68]
Larval zebrafish	Polystyrene microplastics (5μm and 50μm)	● Altered abundance and diversity of the gut microbial community<br>● Changes in metabolic profile, and differential metabolites involved in energy metabolism, glucolipid metabolism, inflammatory response, neurotoxic response, nucleic acid metabolism, and oxidative stress<br>● Hydrogen peroxidase activity and GSH content were significantly reduced<br>● Changes in genes related to glycolysis and lipid metabolism	[25]
Fathead minnow （*pimephalespromelas*）	Polystyrene nanoplastics (41nm), Polycarbonate nanoplastics (158.7nm)	● Significantly increased degranulation of primary granules and release of neutrophil extracellular traps<br>● Interfere with the disease resistance of fish populations	[72]
African catfish (*Clarias gariepinus*)	Polystyrene microplastics (<60μm)	● Significant increase in the degree of liver tissue changes<br>● Significantly reduced transcript levels of tryptophan hydroxylase 2	[73]

	in brain tissue<br>● Regulation of gill tissue changes, plasma cholesterol, HDL, total protein, albumin and globulin concentrations, liver glycogen reserves, and transcript levels of Fushitarazu factor 1, Gonadotropin-releasing hormone, and 11β-hydroxysteroid dehydrogenase type 2

50. de Souza Machado, A.A.; Lau, C.W.; Till, J.; Kloas, W.; Lehmann, A.; Becker, R.; Rillig, M.C. Impacts of Microplastics on the Soil Biophysical Environment. *Environ Sci Technol* **2018**, *52*, 9656-9665, doi:10.1021/acs.est.8b02212.

10. Liu, H.; Yang, X.; Liu, G.; Liang, C.; Xue, S.; Chen, H.; Ritsema, C.J.; Geissen, V. Response of soil dissolved organic matter to microplastic addition in Chinese loess soil. *Chemosphere* **2017**, *185*, 907-917, doi:10.1016/j.chemosphere.2017.07.064.

51. López-Rojo, N.; Pérez, J.; Alonso, A.; Correa-Araneda, F.; Boyero, L. Microplastics have lethal and sublethal effects on stream invertebrates and affect stream ecosystem functioning. *Environ Pollut* **2020**, *259*, 113898, doi:10.1016/j.envpol.2019.113898.

52. Green, D.S.; Jefferson, M.; Boots, B.; Stone, L. All that glitters is litter? Ecological impacts of conventional versus biodegradable glitter in a freshwater habitat. *J Hazard Mater* **2021**, *402*, 124070, doi:10.1016/j.jhazmat.2020.124070.

53. Wang, J.; Lv, S.; Zhang, M.; Chen, G.; Zhu, T.; Zhang, S.; Teng, Y.; Christie, P.; Luo, Y. Effects of plastic film residues on occurrence of phthalates and microbial activity in soils. *Chemosphere* **2016**, *151*, 171-177, doi:10.1016/j.chemosphere.2016.02.076.

54. Bosker, T.; Bouwman, L.J.; Brun, N.R.; Behrens, P.; Vijver, M.G. Microplastics accumulate on pores in seed capsule and delay germination and root growth of the terrestrial vascular plant Lepidium sativum. *Chemosphere* **2019**, *226*, 774-781, doi:10.1016/j.chemosphere.2019.03.163.

55. Nolte, T.M.; Hartmann, N.B.; Kleijn, J.M.; Garnæs, J.; van de Meent, D.; Jan Hendriks, A.; Baun, A. The toxicity of plastic nanoparticles to green algae as influenced by surface modification, medium hardness and cellular adsorption. *Aquat Toxicol* **2017**, *183*, 11-20, doi:10.1016/j.aquatox.2016.12.005.

56. Zhang, C.; Chen, X.; Wang, J.; Tan, L. Toxic effects of microplastic on marine microalgae Skeletonema costatum: Interactions between microplastic

and algae. *Environ Pollut* **2017**, *220*, 1282-1288, doi:10.1016/j.envpol.2016.11.005.
57. Zhou, Y.; Liu, X.; Wang, J. Ecotoxicological effects of microplastics and cadmium on the earthworm *Eisenia foetida*. *J Hazard Mater* **2020**, *392*, 122273, doi:10.1016/j.jhazmat.2020.122273.
58. Jiang, X.; Chang, Y.; Zhang, T.; Qiao, Y.; Klobučar, G.; Li, M. Toxicological effects of polystyrene microplastics on earthworm (*Eisenia fetida*). *Environ Pollut* **2020**, *259*, 113896, doi:10.1016/j.envpol.2019.113896.
59. Tlili, S.; Jemai, D.; Brinis, S.; Regaya, I. Microplastics mixture exposure at environmentally relevant conditions induce oxidative stress and neurotoxicity in the wedge clam *Donax trunculus*. *Chemosphere* **2020**, *258*, 127344, doi:10.1016/j.chemosphere.2020.127344.
60. Sussarellu, R.; Suquet, M.; Thomas, Y.; Lambert, C.; Fabioux, C.; Pernet, M.E.J.; Le Goïc, N.; Quillien, V.; Mingant, C.; Epelboin, Y.; et al. Oyster reproduction is affected by exposure to polystyrene microplastics. *Proc Natl Acad Sci U S A* **2016**, *113*, 2430-2435, doi:10.1073/pnas.1519019113.
21. Pitt, J.A.; Kozal, J.S.; Jayasundara, N.; Massarsky, A.; Trevisan, R.; Geitner, N.; Wiesner, M.; Levin, E.D.; Di Giulio, R.T. Uptake, tissue distribution, and toxicity of polystyrene nanoparticles in developing zebrafish (*Danio rerio*). *Aquat Toxicol* **2018**, *194*, 185-194, doi:10.1016/j.aquatox.2017.11.017.
61. Pitt, J.A.; Trevisan, R.; Massarsky, A.; Kozal, J.S.; Levin, E.D.; Di Giulio, R.T. Maternal transfer of nanoplastics to offspring in zebrafish (*Danio rerio*): A case study with nanopolystyrene. *Sci Total Environ* **2018**, *643*, 324-334, doi:10.1016/j.scitotenv.2018.06.186.
62. Bourdages, M.P.T.; Provencher, J.F.; Baak, J.E.; Mallory, M.L.; Vermaire, J.C. Breeding seabirds as vectors of microplastics from sea to land: Evidence from colonies in Arctic Canada. *Sci Total Environ* **2021**, *764*, 142808, doi:10.1016/j.scitotenv.2020.142808.
63. Provencher, J.F.; Gaston, A.J.; Mallory, M.L.; O'Hara, P.D.; Gilchrist, H.G. Ingested plastic in a diving seabird, the thick-billed murre (*Uria lomvia*), in the eastern Canadian Arctic. *Mar Pollut Bull* **2010**, *60*, 1406-1411, doi:10.1016/j.marpolbul.2010.05.017.
64. Xia, X.; Sun, M.; Zhou, M.; Chang, Z.; Li, L. Polyvinyl chloride microplastics induce growth inhibition and oxidative stress in Cyprinus carpio var. larvae. *Sci Total Environ* **2020**, *716*, 136479, doi:10.1016/j.scitotenv.2019.136479.
23. Lu, Y.; Zhang, Y.; Deng, Y.; Jiang, W.; Zhao, Y.; Geng, J.; Ding, L.; Ren, H. Uptake and Accumulation of Polystyrene Microplastics in Zebrafish (*Danio rerio*) and Toxic Effects in Liver. *Environ Sci Technol* **2016**, *50*, 4054-4060, doi:10.1021/acs.est.6b00183.
65. Wang, J.; Li, Y.; Lu, L.; Zheng, M.; Zhang, X.; Tian, H.; Wang, W.; Ru, S. Polystyrene microplastics cause tissue damages, sex-specific reproductive disruption and transgenerational effects in marine medaka (*Oryzias melastigma*). *Environ Pollut* **2019**, *254*, 113024, doi:10.1016/j.envpol.2019.113024.
66. Qiao, R.; Sheng, C.; Lu, Y.; Zhang, Y.; Ren, H.; Lemos, B. Microplastics induce intestinal inflammation, oxidative stress, and disorders of metabolome and microbiome in zebrafish. *Sci Total Environ* **2019**, *662*, 246-253, doi:10.1016/j.scitotenv.2019.01.245.
67. Lei, L.; Wu, S.; Lu, S.; Liu, M.; Song, Y.; Fu, Z.; Shi, H.; Raley-Susman, K.M.; He, D. Microplastic particles cause intestinal damage and other adverse

effects in zebrafish *Danio rerio* and nematode *Caenorhabditis elegans*. *Sci Total Environ* **2018**, *619-620*, 1-8, doi:10.1016/j.scitotenv.2017.11.103.

68. Qiang, L.; Cheng, J. Exposure to microplastics decreases swimming competence in larval zebrafish (*Danio rerio*). *Ecotoxicol Environ Saf* **2019**, *176*, 226-233, doi:10.1016/j.ecoenv.2019.03.088.

25. Wan, Z.; Wang, C.; Zhou, J.; Shen, M.; Wang, X.; Fu, Z.; Jin, Y. Effects of polystyrene microplastics on the composition of the microbiome and metabolism in larval zebrafish. *Chemosphere* **2019**, *217*, 646-658, doi:10.1016/j.chemosphere.2018.11.070.

72. Greven, A.-C.; Merk, T.; Karagöz, F.; Mohr, K.; Klapper, M.; Jovanović, B.; Palić, D. Polycarbonate and polystyrene nanoplastic particles act as stressors to the innate immune system of fathead minnow (*Pimephales promelas*). *Environ Toxicol Chem* **2016**, *35*, 3093-3100, doi:10.1002/etc.3501.

73. Karami, A.; Romano, N.; Galloway, T.; Hamzah, H. Virgin microplastics cause toxicity and modulate the impacts of phenanthrene on biomarker responses in African catfish (*Clarias gariepinus*). *Environ Res* **2016**, *151*, 58-70, doi:10.1016/j.envres.2016.07.024.
